# The minimal important change for the EQ VAS based on the SF-36 health transition item: observations from 25772 spine surgery procedures

**DOI:** 10.1007/s11136-022-03182-3

**Published:** 2022-07-11

**Authors:** Anders Joelson, Fredrik Nerelius, Freyr Gauti Sigmundsson, Jan Karlsson

**Affiliations:** 1 Department of Orthopedics, Orebro University School of Medical Sciences and Orebro University Hospital, SE70185 Orebro, Sweden; 2 Faculty of Medicine and Health, University Health Care Research Center, Orebro University, SE70182 Orebro, Sweden

**Keywords:** Disk herniation, EQ-5D, EQ VAS, Health transition item, MCID, MIC, ROC, SF-36, Spinal stenosis

## Abstract

**Purpose:**

The EQ VAS is an integral part of EQ-5D, a commonly used instrument for health-related quality of life assessment. This study aimed to calculate the minimal important change (MIC) thresholds for the EQ VAS for improvement and deterioration after surgery for disk herniation or spinal stenosis.

**Methods:**

Patients, who were surgically treated for disk herniation or spinal stenosis between 2007 and 2016, were recruited from the Swedish spine register. Preoperative and 1-year postoperative data for a total of 25772 procedures were available for analysis. We used two anchor-based methods to estimate MIC for EQ VAS: (1) a predictive model based on logistic regression and (2) receiver operating characteristics (ROC) curves. The SF-36 health transition item was used as anchor.

**Results:**

The EQ VAS MIC threshold for improvement after disk herniation surgery ranged from 8.25 to 11.8 while the corresponding value for deterioration ranged from − 6.17 to 0.5. For spinal stenosis surgery the corresponding MIC values ranged from 10.5 to 14.5 and − 7.16 to − 6.5 respectively. There were moderate negative correlations (disk herniation − 0.47, spinal stenosis − 0.46) between the 1 year change in the EQ VAS and the SF-36 health transition item (MIC anchor).

**Conclusions:**

For EQ VAS, we recommend a MIC threshold of 12 points for improvement after surgery for disk herniation or spinal stenosis, whereas the corresponding threshold for deterioration is − 7 points. There are marked differences between the EQ VAS MIC for improvement and deterioration after surgery for disk herniation or spinal stenosis. The MIC value varied depending on the method used for MIC estimation.

**Supplementary Information:**

The online version contains supplementary material available at 10.1007/s11136-022-03182-3.

## Introduction

Values for the minimal important change (MIC) have become increasingly important in the era of large-scale register-based research because clinically irrelevant changes may become statistically significant due to large sample sizes [1]. Several different concepts for the minimal important change are used interchangeably: minimal important change (MIC), minimal/minimum clinically important difference (MCID), and minimal clinically important improvement (MCII). In this paper we define MIC as the smallest difference in score in the domain of interest that patients perceive as beneficial (i.e. the definition of MCID used by Jaeschke et al. [2]). MIC values are used to evaluate changes within a group, e.g., before and after a medical intervention. In contrast, the minimal important difference (MID) is used to evaluate differences between groups [3]. An equally important concept is the minimal/smallest detectable change (MDC/SDC) (also called the smallest real difference, SRD) which is the smallest measurement change, that can be interpreted as a real difference (i.e., not a measurement error) [4]. The concept of MIC is controversial and there are concerns that clinical importance is not adequately captured by MIC values [5].

Historically, there have been two major methodological approaches to determine MIC values: (1) distribution-based methods, and (2) anchor-based methods [6]. Terwee et al. [7], in a conceptual clarification, questioned the use of distribution-based methods because these methods evaluate measurement errors (e.g. MDC) but do not relate to the importance of change. However, information about the measurement error is important for assessing the quality of the measurement. If the measurement error is larger than the MIC, measures should be taken to reduce the measurement error in order to evaluate the MIC [8].

Studies on MIC often focus on the minimal important improvement. The rationale for this is that MIC values are commonly used to assess the effects of medical interventions aimed at improving health. However, the minimal important deterioration is equally important. One approach to assess deterioration is to simply use the MIC for improvement but with the opposite sign. However, previous studies have reported differences in the magnitude between MIC for improvement and MIC for deterioration. For example, based on data from the Norwegian registry for spine surgery, Werner et al. [9] report different MIC cutoff values for failure for common PROMs used in spine surgery compared to the corresponding values for success reported by Solberg et al. [10].

Elective spine surgery aims to reduce pain and disability. Consequently, spine surgery outcome measures focus on pain and disability measurements. Commonly used outcome measures are numeric rating scales (NRS) for back and/or leg pain and disease-specific disability measures such as the Oswestry disability index. Previous studies have reported the MIC values of these outcome measures [9, 10]. The MIC values can be used in clinical practice to inform patients about the expected effects of surgical procedures, e.g. the percentage of patients who experience a minimal important change after a given surgical procedure [7]. Equally important is the assessment of general health-related quality of life (HRQoL) after spine surgery. The EQ-5D index [11] is a commonly used instrument for health-related quality of life (HRQoL) assessment which is also used to evaluate medical interventions from an economic perspective.

The EQ VAS is an integral part of EQ-5D. Surprisingly few investigations have evaluated the MIC for the EQ VAS in orthopedic conditions [6, 12]. In this study, we used data from the Swedish spine register, Swespine, to calculate anchor-based MIC values (improvement and deterioration) for the EQ VAS for the two common spine surgery procedures, disk herniation surgery and spinal stenosis surgery.

## Patients and methods

### Study design

The present study was a register study based on prospectively collected longitudinal data from Swespine, the national Swedish spine register.

### The national Swedish spine register (Swespine)

Swespine was launched in 1992, the coverage is 90% of the spine units in Sweden and the follow-up rate is 75–80% [13]. The register includes data on diagnoses, surgical procedures, complications, and PROMs after 1, 2, 5, and 10 years. The surgeon is responsible for submitting data about the surgery, whereas the patient submits background data and completes the PROM forms. The Swespine office organizes the follow-up and the surgeons are not involved. The forms are completed digitally or on paper. Participation is voluntary for the patients (opt-out is used) and can be withdrawn at any time.

### Measures

SF-36 is an eight-dimensional, 36-item, self-administered HRQoL instrument for the assessment of general HRQoL [14]. We used the Swedish translation of SF-36 version one [15]. Item two of SF-36 is a health transition item with five response options coded on an ordinal scale from one to five, one being the best and five the worst (Table S1). In a previous study we found that SF-36 item two was a responsive measure of self-rated general health when evaluating surgical outcome in spine surgery [16]. We used the SF-36 item two as anchor in our MIC estimation.

The EQ-5D is a self-administered HRQoL instrument for the assessment of general HRQoL [11]. The current study used the three-level version of the EQ-5D. The instrument includes a 20 cm vertical visual analogue scale (EQ VAS), graded 0–100, (0 being the worst imaginable health state and 100 being the best imaginable health state) for assessment of general health.

### Patient data set

Patient data were retrieved from Swespine. A total of 46658 surgical procedures for treatment of lumbar spinal stenosis or lumbar disk herniation between 2007 and 2016 are included in the register. Preoperative or 1-year postoperative SF-36, or EQ VAS data were incomplete for 20886 procedures, yielding 25772 procedures eligible for analysis (disk herniation 10358 procedures, spinal stenosis 15414 procedures). All patients with incomplete data were excluded from the data analysis. The characteristics of the study population are shown in Table 1. The characteristics of the excluded patients are presented in Table S2.

**Table 1:** Characteristics of the study population

Parameter	Disk herniation	Spinal stenosis
*n*	10358	15414
Age, mean (SD)	46.3 (13.8)	68.2 (10.2)
BMI, mean (SD)	26.4 (4.2)	27.7 (4.2)
Women, *n* (%)	4818 (46.5)	7308 (47.4)
EQ VAS, mean (SD)	45.3 (22.4)	49.3 (21.9)

### MIC estimation

We used two anchor-based methods to estimate the MIC for EQ VAS: a predictive model based on logistic regression [17] and receiver operating characteristics (ROC) curves [18]. The SF-36 health transition item (item two) was used as anchor. We used the pROC package [19] for R (R Foundation for Statistical Computing, Vienna, Austria, 2017) for the ROC analysis. We used two criteria for estimating the MIC: (1) the point on the ROC curve closest to the top left corner of the ROC plot (i.e. minimum of (1-specificity)^2^ + (1-sensitivity)^2^) and (2) the maximum Youden index [20, 21]. The area under the ROC curve (AUC) was calculated as a measure of discriminative ability, AUC > 0.70 was considered acceptable [3, 22]. The anchor should measure essentially the same latent variable as the target instrument. Revicki et al. [23] recommend 0.30–0.35 as a correlation threshold. The textbook by Fayers et al. [24] recommend a correlation threshold of 0.375.

### Definition of improvement, no change, and deterioration

The SF-36 health transition item (item two) was used to define improvement, no change, and deterioration. Patients reporting *much better* or *somewhat better* (response options one or two) were classified as improved, patients reporting about the same (response option three) were classified as *unchanged*, and reporting *somewhat wors*e or *much worse* (response options four or five) were classified as deteriorated.

### Statistics

Continuous data are presented as mean and standard deviation (SD) and/or 95% confidence intervals (CIs). Categorical data are presented as numbers and percentages. Bootstrapping was used to calculate CIs [25]. Standardized response mean (SRM) for paired data, i.e. the difference in means divided by the standard deviation of the difference, was used to evaluate effect size. The SRM was interpreted as follows: <0.2 no effect, 0.2 to 0.4 small effect, 0.5 to 0.7 moderate effect, >0.7 large effect [24]. We used kernel density estimation with Gaussian kernels to estimate the EQ VAS distributions (R Foundation for Statistical Computing, Vienna, Austria, 2017). The Spearman rank coefficient was used to investigate correlations between the 1-year change in the EQ VAS and the SF-36 health transition item. The strength of a correlation was interpreted as follows: 0.10 to 0.29 as small, 0.30 to 0.49 as medium and 0.50–1.0 as large [26].

## Results

**Fig. 1:**
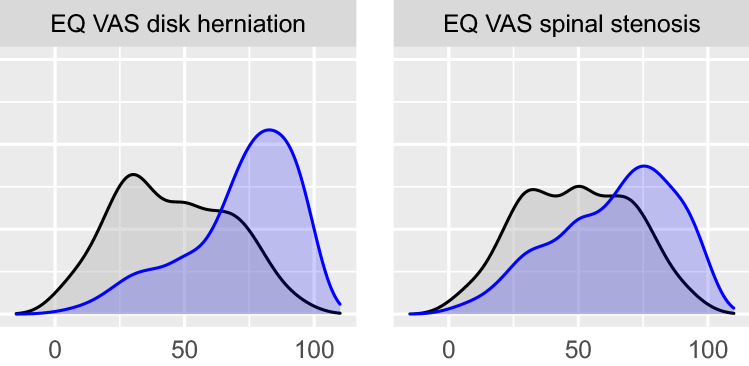
EQ VAS distribution for disk herniation (*n*=10358) and spinal stenosis (*n*=15414) preoperatively (black) and one year postoperatively (blue). (Color figure online)

**Fig. 2:**
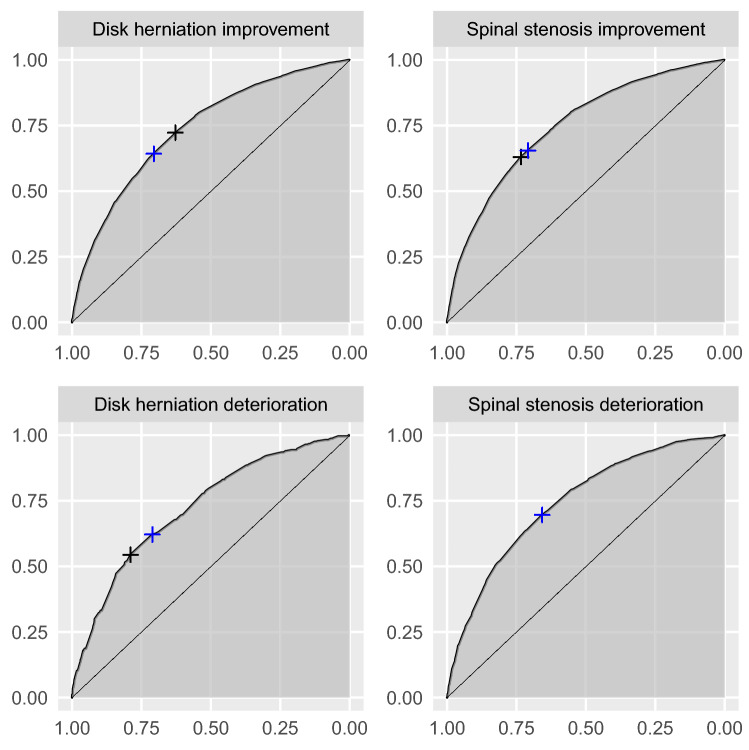
ROC curves for EQ VAS for disk herniation (*n*=10358) and spinal stenosis (*n*=15414) using the SF-36 transition item as anchor. Blue cross: the point closest to the top left corner. Black cross: the maximum Youden Index. (Color figure online)

The preoperative and 1-year postoperative EQ VAS distributions are shown in Figure 1. The number of improved, unchanged, and deteriorated patients are presented in Tables 2 and S3 respectively. The effect sizes of change (SRMs) were large for improvement for both disk herniation and spinal stenosis whereas SRMs for deterioration were <0.2 (no effect). Table 3 summarizes MIC thresholds for improvement and deterioration for disk herniation and spinal stenos. The ROC curves for improvement and deterioration for disk herniation and spinal stenosis are given in Figure 2. The ROC analysis showed some variation in the thresholds whereas the regression thresholds were more uniform. Based on the results presented in Table 3, we recommend the MIC threshold 12 points for improvement after surgery for disk herniation or spinal stenosis. For deterioration after surgery for disk herniation or spinal stenosis we recommend the MIC threshold -7 points.

**Table 2:** EQ VAS results for improvement (SF-36 item two, response option one and two), no change (SF-36 item two, response option three) and deterioration (SF-36 item two, response option four and five) at year one

	Improvement	No change	Deterioration
Disk herniation			
* n* (%)	8290 (80)	1318 (12.7)	750 (7.24)
Difference, mean (CI)	31.5 (30.9; 32)	8.47 (7.18; 9.8)	0.044 (− 1.79; 1.79)
SRM, mean (CI)	1.22 (1.19; 1.25)	0.342 (0.287; 0.398)	0.00171 (− 0.0699; 0.0733)
Spinal stenosis			
*n* (%)	9503 (61.7)	3383 (21.9)	2528 (16.4)
Difference, mean (CI)	23 (22.5; 23.5)	4.75 (4.04; 5.45)	− 3.55 (− 4.52; − 2.61)
SRM, mean (CI)	0.957 (0.933; 0.982)	0.217 (0.183; 0.251)	− 0.147 (− 0.186; − 0.108)

**Table 3:** MIC values for EQ VAS for improvement and deterioration for disk herniation (*n*=10358) and spinal stenosis (*n*=15414)

	Threshold Youden	Sensitivity Youden	Specificity Youden	Threshold Top left	Sensitivity Top left	Specificity Top left	AUC	Regression model
Improvement								
Disk herniation, mean (CI)	8.25 (3.5; 13.5)	0.723 (0.619; 0.788)	0.628 (0.563; 0.731)	10.5 (8.25; 11.5)	0.642 (0.632; 0.731)	0.706 (0.623; 0.718)	0.736 (0.726; 0.746)	11.8 (10.5; 13)
Spinal stenosis, mean (CI)	14.5 (5.5; 14.5)	0.63 (0.623; 0.76)	0.734 (0.604; 0.743)	10.5 (10.5; 12.5)	0.655 (0.637; 0.669)	0.708 (0.69; 0.728)	0.746 (0.738; 0.755)	12.2 (11.3; 13.2)
Deterioration								
Disk herniation, mean (CI)	0.5 (− 12.5; 5.5)	0.544 (0.497; 0.805)	0.789 (0.527; 0.833)	− 0.5 (− 5.5; − 0.5)	0.621 (0.58; 0.687)	0.711 (0.63; 0.733)	0.723 (0.697; 0.75)	− 6.17 (− 9.66; − 2.81)
Spinal stenosis, mean (CI)	− 6.5 (− 12.5; − 0.5)	0.696 (0.606; 0.802)	0.657 (0.553; 0.74)	− 6.5 (− 8.5; − 2.5)	0.696 (0.628; 0.721)	0.657 (0.645; 0.728)	0.741 (0.725; 0.757)	− 7.16 (− 9.19; − 5.17)

The percentages of patients who reach the improvement and deterioration thresholds are shown in Table S4. Spearman's rank correlations between the EQ VAS and the SF-36 health transition item are shown in Table 4.

**Table 4:** Spearman rank correlations between SF-36 item two and EQ VAS preoperatively, year one after surgery and change (difference year one and preop) for disk herniation (*n*=10358) and spinal stenosis (*n*=15414)

	EQ VAS preop	EQ VAS year one	EQ VAS difference
Disk herniation			
SF-36 item two, preop	− 0.36	− 0.05	0.26
SF-36 item two, year one	− 0.0079	− 0.59	− 0.47
Spinal stenosis			
SF-36 item two, preop	− 0.37	− 0.15	0.18
SF-36 item two, year one	− 0.11	− 0.65	− 0.48

## Discussion

In the present study, we report the MIC values for improvement and deterioration 1 year after surgery for disk herniation and spinal stenosis. Our MIC values were similar to the previously reported EQ VAS MIC values for orthopedic conditions. Soer et al. [27] reported an EQ VAS MIC value of 10.5 points when studying effects of rehabilitation for low back pain (*n*=151). Paulsen et al. [28] reported an EQ VAS MIC value of 23 points when using a disease specific anchor in patients surgically treated with total hip arthroplasty for hip osteoarthritis (*n*=1335). The correlation between the anchor and EQ VAS, however, was weak. Paulsen et al. [28] reported a MIC value of 12 points for a general health change anchor. The correlation between the anchor and the EQ VAS was 0.35 but the ROC AUC was only 0.60. This illustrates the importance of detailed knowledge of MIC validation (type of anchor, anchor-PROM correlation, AUC, sample sizes etc.) when using specific MIC values in clinical trials.

To the best of our knowledge, there are no previous reports on EQ VAS MIC for deterioration after spine surgery. Werner et al. [9] reported MIC values for several commonly used patient reported outcome measures (PROMs) (EQ-5D index, the Oswestry disability index, and numeric rating scales for leg and back pain) for failure after disk herniation surgery. A general health transition item was used as anchor. Interestingly, the MIC values were greater than zero which means that the PROMs of the patients improved but the health transition item showed a health deterioration. In contrast, we report negative MIC values for deterioration in EQ VAS. A possible explanation for this difference is that Werner et al. [9] include patients reporting *no change* in the definition of failure whereas we exclude patients reporting *about the same* (response option three) in our definition of deterioration. Again, this illustrates the importance of detailed knowledge of the anchor when using anchor-based MIC values.

We found a marked difference between the MIC value for improvement and the MIC value for deterioration. One explanation for the difference in MIC for improvement and deterioration might be that there is an imbalance in the distribution of the answers to the SF-36 health transition item between the improved and deteriorated patients (Table S3). For example, for disk herniation surgery, the answers for improvement in health are shifted towards better health (61% *much better* vs. 19% *somewhat better*), which means that the *much better* group contributes more to the MIC than the *somewhat better* group, which results in a high MIC value. In addition, for deterioration after disk herniation surgery, the answers are shifted towards *better* health (4.8% *somewhat worse* vs. 2.4% *much worse*), which resulted in a *lower* MIC value for deterioration. Consequently, because the properties of the distribution of anchor response options (e.g., skewness) will affect the MIC values, detailed knowledge of the anchor distribution is essential when calculating MIC values.

An essential part of the MIC ROC analysis is to determine the optimal threshold for the MIC. We used two optimization criteria for the estimation of the MIC: (1) the point on the ROC curve closest to the top left corner of the ROC plot and (2) the maximum Youden index. Our analysis yielded inconsistent results for these methods (Table 3 and Figure 2). Perkins et al. [21] argued for the use of the maximum Youden index when the results of the two methods were inconsistent.

Additionally, the ROC analysis and the logistic regression model gave inconsistent results (Table 3). The most pronounced differences were observed in deterioration after surgery for disk herniation. Telurin et al. [17] argued in favor of using the logistic regression to determine MIC since MIC estimation based on logistic regression models appears to give smaller variance for the MIC estimate.

When our suggested MIC values for improvement and deterioration after surgery for disk herniation or spinal stenosis (12 and − 7) were applied to our data (Table S4) we found that the percentage of improved patients was lower (68.4% vs. 80%) and the percentage of deteriorated patients was higher (10.4% vs. 7.2%) than the corresponding percentages for the SF-36 health transition item (Table 2). Consequently, our suggested EQ VAS thresholds provides a more conservative estimate of the benefit with regards to general health perceptions after surgery for disk herniation or spinal stenosis compared to the SF-36 health transition item. Guyatt et al. [29] reported that transition ratings might be biased by the current health state. Our data confirm this finding (Table 2). This means that transition ratings may overestimate the effect of a surgical intervention which might be a part of the explanation for the difference between Tables 2 and S4.

The correlation between the anchor and the 1-year change in EQ VAS was -0.47 for disk herniation surgery and − 0.48 for spinal stenosis surgery (Table 4). Revicki et al. [23] recommend 0.30–0.35 as a correlation threshold to define an acceptable association between an anchor and the PROM change score. In contrast, Guyatt et al. [29], have a more restrictive approach and recommend a correlation threshold of 0.50 points. Since there is no consensus regarding correlation thresholds, and also because our correlations are in the upper region of the medium correlation range proposed by Cohen [26], we find it reasonable to use the SF-36 transition item as anchor for MIC calculations for the EQ VAS.

SF-36 provides alternative measures that could be used as anchors for MIC calculation: SF-36 item one (the single item for self-rated health assessment, SRH) and the general health (GH) domain. In our prior work on HRQoL, however, we noted that the responsiveness to change after spine surgery for SF-36 item one and the GH domain was limited, which makes these measures less suitable as anchors [16, 30].

The findings of our study should be evaluated in the light of several limitations. First, the data were limited to patients surgically treated for disk herniation or spinal stenosis. Other uses of the MIC values of our study should be made with caution. Second, we recognize the inherent limitations of register data, e.g., lack of confounder information, missing data, or unknown data quality [1]. Third, information about co-morbidities that might affect general heath perceptions were lacking. Fourth, data were incomplete for 20886 (44%) of the procedures. Fifth, we did not evaluate the MDC of EQ VAS. The MIC has to be greater than the MDC to be a valid threshold [8]. Sixth, we did not adjust our MIC values for differences in EQ VAS at baseline. This is recognized as a limitation because previous studies have suggested that differences in baseline PROMs may affect MIC thresholds [9, 31]. Seventh, data on socioeconomic factors were lacking. The study of Iderberg et al. [32] demonstrated that socioeconomic indicators were associated with outcomes of surgery for lumbar spinal stenosis.

Despite these limitations, we believe that the results of our study, are still fairly accurate estimates of the MIC values for EQ VAS and that future studies may now use EQ VAS as a complement to the widely used EQ-5D index in the assessing changes in general HRQoL after spine surgery.

## Conclusion

For the EQ VAS we recommend a MIC threshold of 12 points for improvement after surgery for disk herniation or spinal stenosis whereas the corresponding threshold for deterioration is − 7 points. There are marked differences between the EQ VAS MIC for improvement and deterioration after surgery for disk herniation or spinal stenosis. The MIC value varied depending on the method used for MIC estimation.

## Supplementary Information

Below is the link to the electronic supplementary material.

Supplementary file1 (DOCX 46 KB)

## Data Availability

Data are available from the national Swedish spine register (Swespine) after approval by the Swedish Ethical Review Authority and according to the regulations in the General Data Protection Regulation and the Swedish Patient Data Act.
