# Supplementary material for: The minimal important change for the EQ VAS based on the SF-36 health transition item: observations from 25772 spine surgery procedures
Source: Qual Life Res. 2022 Jul 11;31(12):3459–66. doi: 10.1007/s11136-022-03182-3 (PMC9587963; doi:10.1007/s11136-022-03182-3)
Supplement: Supplementary file 1 — Supplementary file1 (DOCX 46 KB) [file 11136_2022_3182_MOESM1_ESM.docx]

**Table S1** Question and response options for the SF-36 health transition item (item two).

| **Item number** | **Question** | **Response choices** |
| --- | --- | --- |
| SF-36 item two | Compared to one year ago, how would you rate your health in general now? | 1. Much better now than one year ago  2. Somewhat better now than one year ago  3. About the same  4. Somewhat worse now than one year ago  5. Much worse now than one year ago |

**Table S2** Characteristics of the excluded patients.

| **Parameter** | **Disk herniation** | **Spinal stenosis** |
| --- | --- | --- |
| n | 10620 | 10266 |
| Age, mean (SD) | 43.9 (13.6) | 67.9 (11.6) |
| BMI, mean (SD) | 26.6 (4.41) | 27.8 (4.3) |
| Women, n (%) | 4574 (43.1) | 5163 (50.3) |

**Table S3** EQ VAS difference and EQ VAS SRM for the different answers to SF-36 item two year one after surgery for disk herniation (n=10358) and spinal stenosis (n=15414).

|  | **Much better** | **Somewhat better** | **About the same** | **Somewhat worse** | **Much worse** |
| --- | --- | --- | --- | --- | --- |
| **Disk herniation** |  |  |  |  |  |
| n (%) | 6272 (61) | 2018 (19) | 1318 (13) | 498 (4.8) | 252 (2.4) |
| EQ VAS diff, mean (CI) | 36 (36;37) | 17 (16;18) | 8.5 (7.2;9.8) | 1.9 (-0.44;4.2) | -3.7 (-6.8;-0.56) |
| EQ VAS SRM, mean (CI) | 1.5 (1.4;1.5) | 0.71 (0.66;0.76) | 0.34 (0.29;0.4) | 0.076 (-0.012;0.16) | -0.14 (-0.26;-0.014) |
| **Spinal stenosis** |  |  |  |  |  |
| n (%) | 6128 (40) | 3375 (22) | 3383 (22) | 1691 (11) | 837 (5.4) |
| EQ VAS diff, mean (CI) | 28 (28;29) | 13 (12;14) | 4.8 (4;5.5) | -1.2 (-2.3;-0.038) | -8.3 (-10;-6.7) |
| EQ VAS SRM, mean (CI) | 1.2 (1.2;1.3) | 0.59 (0.56;0.63) | 0.22 (0.18;0.25) | -0.052 (-0.099;-0.0041) | -0.32 (-0.39;-0.25) |

**Table S4** Percentage of patients that reaches the MIC improvement threshold 12 and the MIC deterioration threshold -7 for disk herniation and spinal stenosis.

| **Parameter** | **MIC improvement** | **MIC deterioration** |
| --- | --- | --- |
| Disk herniation, n (%) | 7090 (68.4) | 1076 (10.4) |
| Spinal stenosis, n (%) | 7926 (51.4) | 2811 (18.2) |

**Table S5** SF-36 item two year one after surgery for disk herniation (n=10358) and spinal stenosis (n=15414) for patients with complete (preoperative and one-year data) EQ VAS data (n=24817) and incomplete (missing preoperative or one-year data) EQ VAS data (n=8419).

|  | **Much better** | **Somewhat better** | **About the same** | **Somewhat worse** | **Much worse** |
| --- | --- | --- | --- | --- | --- |
| **Complete EQ VAS, n (%)** | 11671 (47%) | 5157 (21%) | 4578 (18%) | 2151 (9%) | 1056 (4%) |
| **Incomplete EQ VAS, n (%)** | 3719 (44%) | 1744 (21%) | 1604 (19%) | 883 (10%) | 519 (6%) |
